# Supplementary material for: Transient cerebral hypoperfusion and hypertensive events during atrial fibrillation: a plausible mechanism for cognitive impairment
Source: Sci Rep. 2016 Jun 23;6:28635. doi: 10.1038/srep28635 (PMC4917883; doi:10.1038/srep28635)
Supplement: Supplementary Information [file srep28635-s1.pdf]

Supplementary Information

Transient cerebral hypoperfusion and hypertensive events during atrial fibrillation: a plausible mechanism for cognitive impairment

M. Anselmino[a], S. Scarsoglio[b], A. Saglietto[a], F. Gaita[a], L. Ridolfi[c]

[a] *Division of Cardiology, Department of Medical Sciences, "Città della Salute e della Scienza" Hospital, University of Turin, Torino, Italy*
[b] *Department of Mechanical and Aerospace Engineering, Politecnico di Torino, Torino, Italy*
[c] *Department of Environmental, Land and Infrastructure Engineering, Politecnico di Torino, Torino, Italy*

Supplementary Methods

Mathematical Modeling

The lumped model, proposed by Ursino and Giannessi (see [11] of the Main Text), extends the Windkessel approach to the arterial and venous cerebral circulation, and is divided into three main parts: large arteries, distal arterial circulation, and capillary-venous circulation. The model is composed by a network of compliances, C, and resistances, R. The viscous effects are taken into account by the resistances, R [mmHg s/ml], while the elastic vessel properties are described by the compliances, C [ml/mmHg]. Three cardiovascular variables are involved at each section: the blood flow, Q [ml/s], the volume, V [ml], the pressure, P [mmHg]. A schematic representation of the cardiovascular system is shown in Fig. 2 of the Main Text.

Equations and Numerical Scheme

For the sake of simplicity, the equations are grouped following the three main partitions of the model. The autoregulation and CO_2 activity equations for the distal district are separately reported.

Large arteries

$$\begin{cases} C_{ICA,left}\left(\dfrac{dP_{MCA,left}}{dt}-\dfrac{dP_{ic}}{dt}\right)=Q_{ICA,left}+Q_{PCoA,left}-Q_{MCA,left}-Q_{ACA1,left}, \\ C_{ICA,right}\left(\dfrac{dP_{MCA,right}}{dt}-\dfrac{dP_{ic}}{dt}\right)=Q_{ICA,right}+Q_{PCoA,right}-Q_{MCA,right}-Q_{ACA1,right}, \\ C_{BA}\dfrac{dP_{BA,willis}}{dt}=Q_{BA}-Q_{PCA1,left}-Q_{PCA1,right}, \end{cases} \tag{S1}$$

$$\begin{cases} Q_{ACA2,left}=Q_{ACA1,left}+Q_{ACoA}, \\ Q_{ACA2,right}=Q_{ACA1,right}-Q_{ACoA}, \\ Q_{PCA2,left}=-Q_{PCoA,left}+Q_{PCA1,left}, \\ Q_{PCA2,right}=-Q_{PCoA,right}+Q_{PCA1,right}, \end{cases} \tag{S2}$$

where the flow rates, Q, are:

$$\begin{cases} Q_{ICA,left}=\dfrac{P_a-P_{MCA,left}}{R_{ICA,left}}, \\ Q_{ICA,right}=\dfrac{P_a-P_{MCA,right}}{R_{ICA,right}}, \\ Q_{BA}=\dfrac{P_a-P_{BA,willis}}{R_{BA}}, \end{cases} \tag{S3}$$

$$\begin{cases} Q_{MCA,left}=\dfrac{P_{MCA,left}-P_{dm,left}}{R_{MCA,left}+R_{dm,left}/2}, \\ Q_{MCA,right}=\dfrac{P_{MCA,right}-P_{dm,right}}{R_{MCA,right}+R_{dm,right}/2}, \\ Q_{ACA1,left}=\dfrac{P_{ICA,left}-P_{ACA,left}}{R_{ACA1,left}}, \\ Q_{ACA1,right}=\dfrac{P_{ICA,right}-P_{ACA,right}}{R_{ACA1,right}}, \\ Q_{PCA1,left}=\dfrac{P_{BA,willis}-P_{PCA,left}}{R_{PCA1,left}}, \\ Q_{PCA1,right}=\dfrac{P_{BA,willis}-P_{PCA,right}}{R_{PCA1,right}}, \end{cases} \tag{S4}$$

$$
\begin{cases}
Q_{ACA2,left} = \dfrac{P_{ACA,left} - P_{da,left}}{R_{ACA2,left} + R_{da,left}/2}, \\
Q_{ACA2,right} = \dfrac{P_{ACA,right} - P_{da,right}}{R_{ACA2,right} + R_{da,right}/2}, \\
Q_{ACoA} = \dfrac{P_{ACA,right} - P_{ACA,left}}{R_{ACoA}}, \\
Q_{PCA2,left} = \dfrac{P_{PCA,left} - P_{dp,left}}{R_{PCA2,left} + R_{dp,left}/2}, \\
Q_{PCA2,right} = \dfrac{P_{PCA,right} - P_{dp,right}}{R_{PCA2,right} + R_{dp,right}/2}, \\
Q_{PCoA,left} = \dfrac{P_{PCA,left} - P_{MCA,left}}{R_{PCoA,left}}, \\
Q_{PCoA,right} = \dfrac{P_{PCA,right} - P_{MCA,right}}{R_{PCoA,right}},
\end{cases}
\tag{S5}
$$

Distal arterial circulation

$$
\begin{cases}
\dfrac{dV_{dm,left}}{dt} = Q_{MCA,left} - Q_{dm,left} + Q_{cam,left} + Q_{cpm,left}, \\
\dfrac{dV_{dm,right}}{dt} = Q_{MCA,right} - Q_{dm,right} + Q_{cam,right} + Q_{cpm,right}, \\
\dfrac{dV_{da,left}}{dt} = Q_{ACA2,left} - Q_{da,left} - Q_{cam,left} + Q_{caa}, \\
\dfrac{dV_{da,right}}{dt} = Q_{ACA2,right} - Q_{da,right} - Q_{cam,right} - Q_{caa}, \\
\dfrac{dV_{dp,left}}{dt} = Q_{PCA2,left} - Q_{dp,left} - Q_{cpm,left} + Q_{cpp}, \\
\dfrac{dV_{dp,right}}{dt} = Q_{PCA2,right} - Q_{dp,right} - Q_{cpm,right} - Q_{cpp},
\end{cases}
\tag{S6}
$$

where the flow rates, Q, are:

$$
\begin{cases}
Q_{dm,left} = \dfrac{P_{dm,left} - P_c}{R_{dm,left}/2}, \\
Q_{dm,right} = \dfrac{P_{dm,right} - P_c}{R_{dm,right}/2}, \\
Q_{da,left} = \dfrac{P_{da,left} - P_c}{R_{da,left}/2}, \\
Q_{da,right} = \dfrac{P_{da,right} - P_c}{R_{da,right}/2}, \\
Q_{dp,left} = \dfrac{P_{dp,left} - P_c}{R_{dp,left}/2}, \\
Q_{dp,right} = \dfrac{P_{dp,right} - P_c}{R_{dp,right}/2},
\end{cases}
\tag{S7}
$$

$$
\begin{cases}
Q_{cam,left} = \dfrac{P_{da,left} - P_{dm,left}}{R_{cam,left}}, \\
Q_{cam,right} = \dfrac{P_{da,right} - P_{dm,right}}{R_{cam,right}}, \\
Q_{cpm,left} = \dfrac{P_{dp,left} - P_{dm,left}}{R_{cpm,left}}, \\
Q_{cpm,right} = \dfrac{P_{dp,right} - P_{dm,right}}{R_{cpm,right}}, \\
Q_{caa} = \dfrac{P_{da,right} - P_{da,left}}{R_{caa}}, \\
Q_{cpp} = \dfrac{P_{dp,right} - P_{dp,left}}{R_{cpp}},
\end{cases}
\tag{S8}
$$

while the constitutive relations are:

$$
\begin{cases}
P_{dm,left} = \dfrac{V_{dm,left}}{C_{dm,left}} + P_{ic}, \\
P_{dm,right} = \dfrac{V_{dm,right}}{C_{dm,right}} + P_{ic}, \\
P_{da,left} = \dfrac{V_{da,left}}{C_{da,left}} + P_{ic}, \\
P_{da,right} = \dfrac{V_{da,right}}{C_{da,right}} + P_{ic}, \\
P_{dp,left} = \dfrac{V_{dp,left}}{C_{dp,left}} + P_{ic}, \\
P_{dp,right} = \dfrac{V_{dp,right}}{C_{dp,right}} + P_{ic},
\end{cases}
\tag{S9}
$$

Capillary-venous circulation

$$
\begin{cases}
C_{ic}\dfrac{dP_{ic}}{dt} = Q_{MCA,left} - Q_{dm,left} + Q_{PCA2,left} - Q_{dp,left} + Q_{ACA2,left} - Q_{da,left} \\
\qquad + Q_{MCA,right} - Q_{dm,right} + Q_{PCA2,right} - Q_{dp,right} + Q_{ACA2,right} - Q_{da,right} \\
\qquad + Q_{pv} - Q_{vs} + Q_f - Q_o, \\
C_{vi}\left(\dfrac{dP_v}{dt} - \dfrac{dP_{ic}}{dt}\right) = Q_{pv} - Q_{vs}, \\
Q_{dm,left} + Q_{da,left} + Q_{dp,left} + Q_{dm,right} + Q_{da,right} + Q_{dp,right} = Q_f + Q_{pv},
\end{cases}
\tag{S10}
$$

where the flow rates, Q, the compliances C_{ic}, C_{vi}, and the resistance R_{vs} are defined as follows:

$$
\begin{cases}
Q_{pv} = \dfrac{P_c - P_v}{R_{pv}}, \\
Q_{vs} = \dfrac{P_v - P_{vs}}{R_{vs}}, \\
Q_f = \begin{cases} \dfrac{P_c - P_{ic}}{R_f}, & \text{if } P_c \geq P_{ic}, \\ 0, & \text{if } P_c < P_{ic}, \end{cases} \\
Q_o = \begin{cases} \dfrac{P_{ic} - P_{vs}}{R_o}, & \text{if } P_{ic} \geq P_{vs}, \\ 0, & \text{if } P_{ic} < P_{vs}, \end{cases} \\
Q_{out} = Q_{vs} + Q_o,
\end{cases}
\tag{S11}
$$

$$
\begin{cases}
C_{ic} = \dfrac{1}{k_E P_{ic}}, \\
C_{vi} = \dfrac{1}{k_{ven}(P_v - P_{ic} - P_{v1})}, \\
R_{vs} = \begin{cases} \dfrac{P_v - P_{vs}}{P_v - P_{ic}} R_{vs1}, & \text{if } P_{vs} < P_{ic}, \\ R_{vs1}, & \text{if } P_{vs} \geq P_{ic}, \end{cases}
\end{cases}
\tag{S12}
$$

Autoregulation and CO_2 reactivity equations

For each of the six distal regions, the following equations hold:

$$
\begin{cases}
\tau_{aut} \dfrac{dx_{aut,i,j}}{dt} = -x_{aut,i,j} + G_{aut} \left(\dfrac{Q_{di,j} - Q_{ndi,j}}{Q_{ndi,j}} \right), & \text{i=m,a,p; j=left,right}, \\
\tau_{CO_2} \dfrac{dx_{CO_2,i,j}}{dt} = -x_{CO_2,i,j} + G_{CO_2} A_{CO_2,i,j} \log_{10} \left(\dfrac{P_{aCO_2}}{P_{aCO_2 n}} \right), & \text{i=m,a,p; j=left,right},
\end{cases}
\tag{S13}
$$

where the subscript n denotes the basal values and

$$
A_{CO_2,i,j} = \frac{1}{1 + \exp\{[-k_{CO_2}(Q_{di,j} - Q_{ndi,j})/Q_{ndi,j}] - b_{CO_2}\}}, \quad \text{i=m,a,p; j=left,right.}
\tag{S14}
$$

Distal compliances and resistances are ruled by the following relations:

$$
\begin{cases}
C_{di,j} = \dfrac{C_{d0i,j} \left[(1 - \Delta C_{di,j}/2) + (1 + \Delta C_{di,j}/2) \exp\left(\frac{x_{CO_2,i,j} - x_{aut,i,j}}{k_{C_{di,j}}} \right) \right]}{1 + \exp[(x_{CO_2,i,j} - x_{aut,i,j})/k_{C_{di,j}}]}, \\
R_{di,j} = \dfrac{k_{R_{di,j}} C^2_{d0i,j}}{V^2_{di,j}}, \quad \text{i=m,a,p; j=left,right},
\end{cases}
\tag{S15}
$$

with

$$
\begin{aligned}
\Delta C_{di,j} &= \begin{cases} 2s_1, & \text{if } x_{CO_2,i,j} < x_{aut,i,j}, \\ 2s_2, & \text{if } x_{CO_2,i,j} \geq x_{aut,i,j}, \end{cases} \quad \text{i=m,a,p; j=left,right} \\
k_{C_{di,j}} &= \begin{cases} \dfrac{C_{d0i,j} s_1}{2}, & \text{if } x_{CO_2,i,j} < x_{aut,i,j}, \\ \dfrac{C_{d0i,j} s_2}{2}, & \text{if } x_{CO_2,i,j} \geq x_{aut,i,j}, \end{cases} \quad \text{i=m,a,p; j=left,right}
\end{aligned}
\tag{S16}
$$

The differential system is numerically solved by means of a multistep adaptative solver, implemented by the `ode15s` Matlab function. This variable order solver is based on the numerical differentiation formulas (NDFs) and is chosen because is one of the most efficient and suitable routines for stiff problems. Indeed, the cerebral differential system shows some stiffness features, that is the equations include some terms that can lead to rapid variation in the solutions. This aspect is particularly evident during end-diastolic and end-systolic phases.

Model Parameters

Parameters of the cerebral model are reported in Tables from S1 to S4.

Parameter	Value
$R_{ICA,left}$	0.5689 mmHg s/ml
$R_{ICA,right}$	0.5689 mmHg s/ml
R_{BA}	0.4501 mmHg s/ml
$R_{MCA,left}$	1.4419 mmHg s/ml
$R_{MCA,right}$	1.4419 mmHg s/ml
$R_{PCA1,left}$	0.7640 mmHg s/ml
$R_{PCA1,right}$	0.7640 mmHg s/ml
$R_{ACA1,left}$	3.7912 mmHg s/ml
$R_{ACA1,right}$	3.7912 mmHg s/ml
$R_{PCA2,left}$	3.6063 mmHg s/ml
$R_{PCA2,right}$	3.6063 mmHg s/ml
$R_{ACA2,left}$	1.6227 mmHg s/ml
$R_{ACA2,right}$	1.6227 mmHg s/ml
$R_{PCoA,left}$	90.9786 mmHg s/ml
$R_{PCoA,right}$	90.9786 mmHg s/ml
R_{ACoA}	14.9228 mmHg s/ml
$C_{ICA,left}$	0.0034 ml/mmHg
$C_{ICA,right}$	0.0034 ml/mmHg
C_{BA}	0.0017 ml/mmHg

Table S1: Large arteries parameters.

Parameter	Value
$R_{cam,left}$	105 mmHg s/ml
$R_{cam,right}$	105 mmHg s/ml
$R_{cpm,left}$	120 mmHg s/ml
$R_{cpm,right}$	120 mmHg s/ml
R_{caa}	22 mmHg s/ml
R_{cpp}	75 mmHg s/ml

Table S2: Distal arterial circulation parameters.

Parameter	Value
R_f	$2.3 \cdot 10^3$ mmHg s/ml
R_o	526.3 mmHg s/ml
R_{pv}	0.880 mmHg s/ml
R_{vs1}	0.366 mmHg s/ml
k_E	0.077 ml^{-1}
k_{ven}	0.155 ml^{-1}
P_{vs}	6 mmHg
P_{v1}	-2.5 mmHg

Table S3: Capillary-venous circulation parameters.

**Parameter**	**Value**
τ_{aut}	20 s
G_{aut}	0.9
τ_{CO_2}	40 s
G_{CO_2}	4.0
k_{CO_2}	15
b_{CO_2}	0.5
P_{aCO_2n}	40 mmHg
P_{aCO_2}	40 mmHg
s_1	7
s_2	0.4
$Q_{ndm,left}$	3.75 ml/s
$Q_{ndm,right}$	3.75 ml/s
$Q_{nda,left}$	1 ml/s
$Q_{nda,right}$	1 ml/s
$Q_{ndp,left}$	1.5 ml/s
$Q_{ndp,right}$	1.5 ml/s
$k_{R_{dm,left}}$	$4.2848 \cdot 10^{\ 4}$ mmHg^{-3} s/ml
$k_{R_{dm,right}}$	$4.2848 \cdot 10^{\ 4}$ mmHg^{-3} s/ml
$k_{R_{da,left}}$	$1.6060 \cdot 10^{\ 5}$ mmHg^{-3} s/ml
$k_{R_{da,right}}$	$1.6060 \cdot 10^{\ 5}$ mmHg^{-3} s/ml
$k_{R_{dp,left}}$	$1.0777 \cdot 10^{\ 5}$ mmHg^{-3} s/ml
$k_{R_{dp,right}}$	$1.0777 \cdot 10^{\ 5}$ mmHg^{-3} s/ml
$C_{d0m,left}$	0.06 ml/mmHg
$C_{d0m,right}$	0.06 ml/mmHg
$C_{d0a,left}$	0.016 ml/mmHg
$C_{d0a,right}$	0.016 ml/mmHg
$C_{d0p,left}$	0.024 ml/mmHg
$C_{d0p,right}$	0.024 ml/mmHg

Table S4: Autoregulation and CO_2 reactivity parameters.

Supplementary Tables

Percentile evaluation of the hemodynamic variables

Table S5 reports the AF percentiles corresponding to the SR thresholds (5% SR and 95% SR) along the ICA-ACA and BA-PCA pathways. The percentile variations are comparable to those observed in the ICA-MCA pathway, as the largest modifications occur downstream of the large artery district. At the venous level, the pressure percentiles modify from 5% (SR) to about 21% (AF), and from 95% (SR) to about 78% (AF). As for the total flow rate return (Q_{out}), the 5% percentile (SR) corresponds to about 24% (AF), while the 95% percentile (SR) to the 77% percentile (AF).

	**AF percentile corresponding to the 5% SR**	**AF percentile corresponding to the 95% SR**
Large arteries		
$P_{BA,willis}$ [mmHg]	16.26	98.70
Q_{BA} [ml/s]	10.45	92.05
$P_{ACA,left}$ [mmHg]	17.47	98.40
$Q_{ACA1,left}$ [ml/s]*	14.02	91.24
$P_{PCA,left}$ [mmHg]	16.41	98.60
$Q_{PCA1,left}$ [ml/s]**	10.65	92.52
Distal arterial circulation		
$P_{da,left}$ [mmHg]	24.16	87.17
$Q_{da,left}$ [ml/s]	14.76	83.43
$P_{dp,left}$ [mmHg]	25.37	90.20
$Q_{dp,left}$ [ml/s]	12.39	85.12
Capillary-venous circulation		
P_v [mmHg]	21.25	78.25
Q_{out} [ml/s]	24.29	77.01

Table S5: AF percentiles corresponding to the SR thresholds (5% SR and 95% SR) along the ICA-ACA and BA-PCA pathways. (*) - (**): in both SR and AF conditions, $Q_{ACA1,left} = Q_{ACA2,left}$ and $Q_{PCA1,left} = Q_{PCA2,left}$.
